# Antioxidant properties of green tea aroma in mice

**DOI:** 10.3164/jcbn.16-80

**Published:** 2017-06-15

**Authors:** Yun-Shan Li, Yuya Kawasaki, Isao Tomita, Kazuaki Kawai

**Affiliations:** 1 Department of Environmental Oncology, Institute of Industrial Ecological Sciences, University of Occupational and Environmental Health, 1-1 Iseigaoka, Yahatanishi-ku, Kitakyushu 807-8555, Japan; 2 School of Pharmaceutical Science, University of Shizuoka, 52-1 Yada, Suruga-ku, Shizuoka 422-8526 Japan

**Keywords:** antioxidant, steam extract condensate of green tea in ethanol (green tea aroma), 8-hydroxydeoxyguanosine (8-OHdG), oxidative stress, X-ray

## Abstract

Green tea (‘Sencha’), made from the leaves of *Camellia sinensis*, is the most well-researched antioxidant beverage. The major source of its antioxidant activity is polyphenols, consisting mainly of catechins (flavan-3-ols). However, little is known about the physiological effects of green tea aroma, which lacks catechins. In the present study, we performed inhalation experiments with green tea aroma to evaluate its antioxidant activity in mice. As a result, the urinary 8-hydroxydeoxyguanosine levels were significantly decreased in comparison with those of the non-treated group, and the serum antioxidant capacity was significantly increased by the inhalation administration of green tea aroma. Furthermore, the increase in the urinary 8-hydroxydeoxyguanosine levels due to whole-body X-ray irradiation was significantly suppressed by the inhalation of green tea aroma. This is the first study to show the antioxidant activity of green tea aroma *in vivo*.

## Introduction

Oxidative stress is considered to be harmful, because reactive oxygen species (ROS) can damage biological molecules, such as lipids, proteins, and DNA. Many studies have suggested that oxidative stress may be involved in the pathophysiology of various diseases, such as diabetes and cancer, as well as aging.^(1)^ Considering these situations, the health benefits of green tea for cancer prevention, as well as its antibacterial and antiviral activities, etc., have been studied for a long time. The beneficial effects of green tea are believed to be mainly caused by the antioxidant properties of catechins, which are present at levels ranging from 8–20% in the tea leaves. Catechins are undoubtedly the major component contributing to the antioxidative properties of green tea, especially its infusions. However, the volatile components of green tea, which have previously been investigated, should also be considered.^(2,3)^ Tea aroma is one of the most important factors in tea preference and quality. The aroma has been used in folk therapy since ancient times, with physiological effects such as tranquilization. With respect to the tea aroma, the essential oil was discovered in 1838. Since then, more than 600 aroma compounds have been chemically identified. However, there is little information about their biological effects. The green tea aroma lacks catechins and their related compounds. In the process of manufacturing green tea, fresh tea leaves are steamed to maintain their green color by enzyme inactivation. At this step, the volatile components can be concentrated within ethanol, as “green tea aroma”. The green tea aroma contains many ingredients composing the fresh scent of early-picked burgeons, including indole, linalool, *cis*-3-hexanol (leaf alcohol) and other components. It lacks non-volatile components, such as tannin. The aroma is a clear, colorless liquid with the fresh scent of a tea plantation. In this study, we evaluated the antioxidant activity of green tea aroma, using mice. We measured the urinary 8-hydroxydeoxyguanosine (8-OHdG) level as the marker for oxidative stress and the serum antioxidant capacity caused by the inhalation of an aromatic volatile concentrate of green tea. 8-OHdG is one of the major oxidation products of nucleobases.^(4)^ It is generated in DNA by various reactive oxygen-forming factors, such as Fenton-type reagents, ionizing radiation, cigarette smoke, and asbestos.^(1)^ As a consequence, it causes gene mutations. Furthermore, 8-OHdG is generated by several repair systems, and is excreted in the urine. For these reasons, the urinary 8-OHdG levels have been widely analyzed as a marker of an individual’s oxidative stress.

## Materials and Methods

### Materials

A steam extract condensate of green tea leaves in ethanol (green tea aroma: Green Breath^®^) was purchased from ACT-FOR Co., Ltd. (Shizuoka, Japan). The green tea aroma was diluted 5-fold with distilled water for administration to the mice. A 20% ethanol solution was used as the control in the animal experiments.

### Animal experiments

#### Chronic administration effect.

Female ICR mice (9 weeks old) were purchased from SLC Japan, Inc. (Shizuoka, Japan). ICR mice (closed colony) are widely used in general toxicity studies, since the diversity of the genetic background, as a closed colony, is valuable for the evaluation of the general toxicity. Ten mice were separated into control and green tea aroma-treated groups (*n* = 5 each). For the green tea aroma-treated group, 1 ml of a 5-fold diluted solution of green tea aroma was sprinkled on the bedding each time it was changed. The mice were moved into new cages every day for the first 3 weeks, and thrice weekly for the 4th through 20th weeks. For the collection of urine, mice were housed in metabolic cages for 24 h at 0, 1, 2, 3, 7, and 20 weeks of inhalation of green tea aroma. Their body weights, dietary intake and water consumption per day were measured at the same time as the urine collection. The serum was collected at 20 weeks of inhalation of green tea aroma or 20% ethanol as the control. The 24 h collected urine and serum at 20 weeks were stored at −20°C until the measurements of urinary 8-OHdG and antioxidant capacity.

#### Radioprotective effect.

Female C57BL/6J mice (8 weeks old) were purchased from SLC Japan, Inc. (Shizuoka, Japan). The C57BL/6J strain (an inbred strain mouse) is commonly used in radiation effect studies, due to the lack of individual genetic differences. Forty mice were separated into two groups: the green tea aroma-treated group (*n* = 20; 5 mice/cage) was treated with 1 ml of a 5-fold diluted solution of green tea aroma every day on the bedding, while the control group (*n* = 20; 5 mice/cage) was treated with 20% ethanol on the bedding. After 1 week of inhalation of green tea aroma or ethanol, each group of mice was separated into four sub-groups. The mice in each sub-group were irradiated with X-rays at 0, 0.25, 0.5 or 0.75 Gy doses. The X-ray irradiation equipment (MBR-1520R; Hitachi, Tokyo, Japan) was operated at 150 kV and 20 mA with a 0.5 mm Al + 0.2 mm Cu filter, at a dose rate of 0.69 Gy/min. The mice were immediately placed into the metabolic cages after the X-ray irradiation for the 24 h urine collection. The serum was collected at 24 h after the X-ray irradiation. The serum and 24 h urine samples were stored at −20°C until analysis.

During the entire investigation, the mice were fed an 8-OHdG-free commercial diet^(5)^ (Dyet no. 110952; Dyets, Inc. Bethlehem, PA) *ad libitum* and given tap water. The mice were kept in a temperature-controlled (25°C) room with a 12 h light/dark cycle during the experimental period. All of the animal experimental procedures and animal handling were performed in accordance with the guidelines described in the Japanese Guide for the Care and Use of Laboratory Animals, as approved by the Animal Care and Use Committee, University of Occupational and Environmental Health, Japan.

### Analysis of urinary 8-OHdG

The urinary 8-OHdG levels were measured according to our previous report,^(5)^ with a slight modification. In brief, a 60 µl aliquot of each urine sample, which was thawed and mixed completely, was mixed with the same volume of a diluting solution containing the ribonucleoside marker 8-hydroxyguanosine. This solution was incubated at 37°C for 40 min, and then centrifuged at 13,000 rpm for 5 min at room temperature. The supernatant was filtered through a pretreatment filter (EKICRODISC, Acro LC3CR, Nihon Pall Ltd., Tokyo, Japan). A 20 µl aliquot of the filtrate was used to analyze the 8-OHdG concentration by the HPLC-ECD method.

### Serum antioxidant capacity

The serum antioxidant capacity was measured with a commercially available kit [Antioxidant Capacity Assay kit (PAO-U), Japan Institute for the Control of Aging (JalCA), Nikken SEIL Co., Ltd., Japan], according to the manufacturer’s instructions.

### Statistical analysis

All data were statistically analyzed using the analysis of variance (ANOVA) and Student *t* test to determine individual differences, using a statistical program package (SPSS, SPSS Inc., Chicago, IL). The data are expressed as the mean ± SD. Statistical significance was assessed as **р<0.01, *p<0.05.

## Results

### Body weight, water intake, and urine output

There were no significant differences in the body weights between the control mice and the green tea aroma-administered mice by inhalation during the experimental period (Table 1). A statistically significant reduction in the amount of drinking water consumption was observed in the green tea aroma inhalation group, even though the urine output was the same as that of the control group.

### Chronic administration effect

**Fig. 1:**
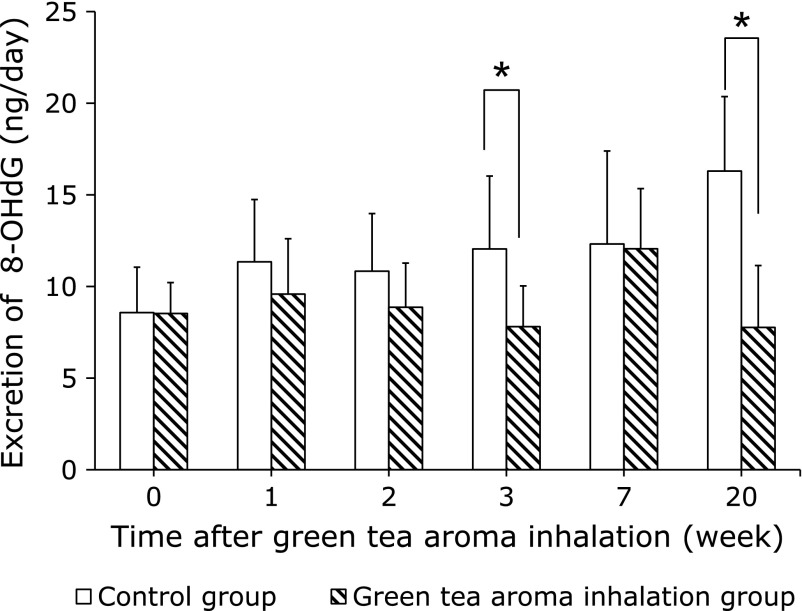
Antioxidant effects of chronic exposure to green tea aroma on the urinary 8-OHdG levels in ICR mice. The urinary 8-OHdG levels are expressed as the amount of 8-OHdG excreted in 24 h, for each mouse. Columns represent mean ± SD (*n* = 5). *p<0.05 (*t* test).

In the ICR mice experiments, the urinary 8-OHdG levels in the green tea aroma groups were lower than those in the control groups (Fig. 1). Although the 8-OHdG levels in the controls seemed to increase with age, those in the green tea aroma group significantly decreased with the duration of the administration, except at 7 weeks. The ratio of the urinary 8-OHdG levels in the green tea aroma group to the control group was decreased by 52% at 20 weeks of inhalation.

### Radioprotective effect

**Fig. 2:**
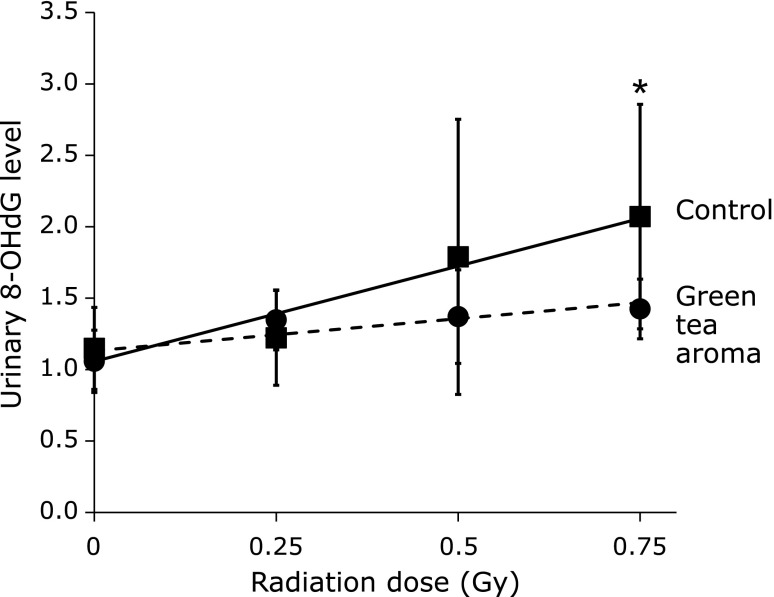
Radioprotective effects of green tea aroma on the urinary levels of 8-OHdG in C57BL/6J mice. The urinary 8-OHdG levels are expressed as the ratio to the original 8-OHdG value of each mouse before the inhalation of green tea aroma or 20% ethanol. Values are mean ± SD (*n* = 3–5). *p<0.05 (ANOVA).

In the experiments with C57BL/6J mice, the urinary 8-OHdG levels were increased dose-dependently by X-ray irradiation (0.25–0.75 Gy) (Fig. 2). In contrast, the increase of the urinary 8-OHdG levels by X-rays was significantly suppressed by the inhalation of green tea aroma.

**Fig. 3:**
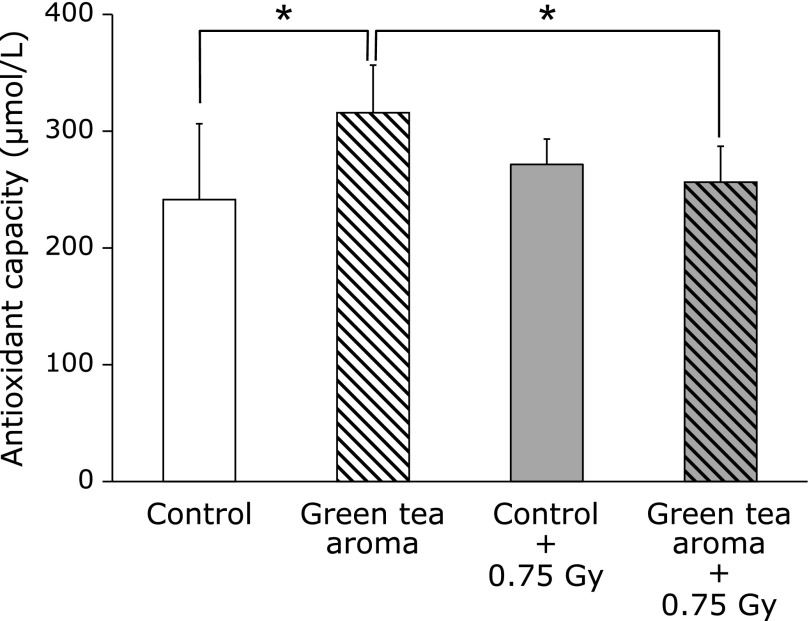
Influence of green tea aroma inhalation on antioxidant capacity in serum. Values are mean ± SD (*n* = 5). *p<0.05 (ANOVA). The serum was collected at 24 h after X-ray irradiation.

Fig. 3 shows the levels of antioxidant capacity in the serum after green tea aroma inhalation. The serum antioxidant capacity in the green tea aroma inhalation group was significantly higher than that in the control group without X-ray irradiation. The levels of the serum antioxidant capacity in the green tea aroma inhalation group were significantly decreased with 0.75 Gy X-ray irradiation.

**Fig. 4:**
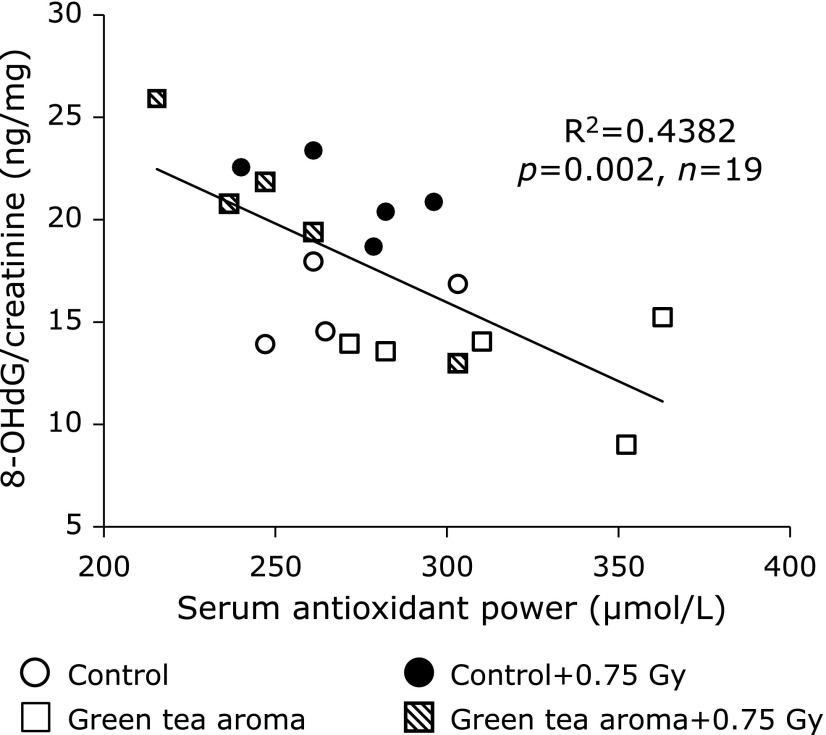
Correlation of urinary 8-OHdG and serum antioxidant capacity in C57BL/6J mice. Urinary 8-OHdG levels and serum antioxidant capacities are plotted for 24 h and for 24 h after X-ray irradiation, respectively.

In addition, a highly significant negative correlation was obtained between the urinary 8-OHdG levels and the serum oxidant capacity levels in C57BL/6J mice (*r* = –0.6619, *p* = 0.002) (Fig. 4).

## Discussions

A significant increase in drinking water consumption upon stress was reported.^(6)^ The reduction in the volume of drinking water by green tea aroma inhalation suggested that the green tea aroma may have tranquilizing effects. Aromatherapy has a long history in Western countries. The aromatic materials are considered to release neurotransmitters with tranquilizing properties. Consequently, they generate an analgesic effect and produce a sense of wellness and relaxation during the therapy. However, the physiological mechanism of aromatherapy with essential oils, including other various aromas, has not been well established yet. Atsumi *et al.*^(7)^ reported that the free radical scavenging activity values in human saliva were increased by a pleasant smell of lavender (*Lavandula angustifolia*) or rosemary (*Rosmarinus officinalis*), while the unpleasant smell of isovarelic acid did not elicit this effect.^(8)^ The smell of coffee has long been known to have an aromatherapeutic effect, and brewed coffee contains many volatile antioxidant compounds.^(9)^ In *C. elegans*, the green tea aroma fraction exhibited antioxidant activity and reduced the effects of beta-amyloid peptide-induced toxicity.^(10)^

The age-related changes in physiological function are considered to be the results of the progressive and irreversible accumulation of oxidative damage. Some studies have reported that the 8-OHdG levels in both urine^(11)^ and tissue DNA increase with age in rodents.^(12)^ Therefore, the urinary 8-OHdG is considered to be a sensitive biomarker for the measurement of oxidative stress and aging. In this study, the age-related increase in the urinary 8-OHdG levels was suppressed by green tea aroma inhalation in mice. Even though there is no statistical difference at 7 weeks, a linear regression analysis of all data revealed a positive correlation between the urinary 8-OHdG levels and the age (in weeks) of the mice in the non-treated group (*p*<0.05), but not in the green tea aroma-treated group. These results indicated that the increased oxidative stress upon aging may be suppressed by green tea aroma inhalation. In addition, the green tea aroma may delay the age-related loss of physiological function caused by oxidative stress.

We previously reported the usefulness of urinary 8-OHdG as a marker for the evaluation of the oxidative stress induced by ionizing radiation. The 8-OHdG levels in urine increased dose-dependently, from about 200 mGy of whole body X-ray irradiation.^(13)^ In this study, X-ray-induced oxidative stress was significantly decreased by green tea aroma inhalation in C57BL/6J mice. The amount of the decrease in the serum antioxidant activity by the X-ray irradiation was parallel to the amount of increase due to green tea aroma. Additionally, there is a good correlation between the urinary 8-OHdG levels and the serum antioxidant capacity. The serum antioxidant capacity induced by green tea aroma worked effectively to reduce the oxidative damage induced by X-ray irradiation in mice. The green tea aroma is expected to be useful as a radioprotective agent.

The antioxidative effects of green tea aroma *in vivo* may be related to the inhalation route of administration, and therefore may not be relevant for antioxidants in orally consumed green tea. The antioxidant mechanism of green tea aroma is still unclear at present. Further studies are needed to determine the relationship between psychological stress and the antioxidant activity induced by green tea aroma inhalation. This is the first report to demonstrate that inhalation exposure to green tea aroma can increase the antioxidant activity and decrease the DNA oxidative damage in mice.

## Figures and Tables

**Table 1:** The characteristics of the animals

Characteristics		Inhalation period of green tea aroma (weeks)					
		0	1	2	3	7	20
Body weight (g)	Control	29.68 ± 0.94	32.12 ± 1.20	31.96 ± 1.60	33.42 ± 1.29	36.88 ± 0.68	48.18 ± 4.54
	Green tea aroma	29.74 ± 0.96	32.38 ± 1.73	33.02 ± 3.27	34.25 ± 2.24	41.02 ± 4.74	55.60 ± 9.14
	Green tea aroma/Control	1	1.01	1.03	1.02	1.11	1.15

Water consumption (ml)	Control	2.52 ± 0.91	1.58 ± 0.96	2.12 ± 0.79	2.30 ± 0.22	1.06 ± 0.28	1.38 ± 0.49
	Green tea aroma	2.52 ± 0.72	1.06 ± 0.36	1.46 ± 0.36	0.96 ± 0.25**	1.16 ± 0.11	0.74 ± 0.47
	Green tea aroma/Control	1	0.67	0.69	0.42	1.09	0.54

Urine (ml)	Control	0.84 ± 0.31	0.82 ± 0.25	0.64 ± 0.13	0.79 ± 0.34	0.62 ± 0.21	0.94 ± 0.41
	Green tea aroma	0.92 ± 0.19	0.82 ± 0.19	0.80 ± 0.36	0.70 ± 0.25	0.95 ± 0.68	0.63 ± 0.52
	Green tea aroma/Control	1.1	1.01	1.26	0.89	1.54	0.67

**p<0.01 (*t* test).

## Abbreviations

8-OHdG: 8-hydroxydeoxyguanosine
*C. elegans*: *Caenorhabditis elegans*
